# DeepAVP-TPPred: identification of antiviral peptides using transformed image-based localized descriptors and binary tree growth algorithm

**DOI:** 10.1093/bioinformatics/btae305

**Published:** 2024-05-06

**Authors:** Matee Ullah, Shahid Akbar, Ali Raza, Quan Zou

**Affiliations:** Institute of Fundamental and Frontier Sciences, University of Electronic Science and Technology of China, Chengdu, Sichuan 610054, China; Institute of Fundamental and Frontier Sciences, University of Electronic Science and Technology of China, Chengdu, Sichuan 610054, China; Department of Computer Science, Abdul Wali Khan University Mardan, Mardan 23200, Pakistan; Department of Computer Science, MY University, Islamabad 45750, Pakistan; Institute of Fundamental and Frontier Sciences, University of Electronic Science and Technology of China, Chengdu, Sichuan 610054, China; Yangtze Delta Region Institute (Quzhou), University of Electronic Science and Technology of China, Quzhou, Zhejiang 324003, China

## Abstract

**Motivation:**

Despite the extensive manufacturing of antiviral drugs and vaccination, viral infections continue to be a major human ailment. Antiviral peptides (AVPs) have emerged as potential candidates in the pursuit of novel antiviral drugs. These peptides show vigorous antiviral activity against a diverse range of viruses by targeting different phases of the viral life cycle. Therefore, the accurate prediction of AVPs is an essential yet challenging task. Lately, many machine learning-based approaches have developed for this purpose; however, their limited capabilities in terms of feature engineering, accuracy, and generalization make these methods restricted.

**Results:**

In the present study, we aim to develop an efficient machine learning-based approach for the identification of AVPs, referred to as DeepAVP-TPPred, to address the aforementioned problems. First, we extract two new transformed feature sets using our designed image-based feature extraction algorithms and integrate them with an evolutionary information-based feature. Next, these feature sets were optimized using a novel feature selection approach called binary tree growth Algorithm. Finally, the optimal feature space from the training dataset was fed to the deep neural network to build the final classification model. The proposed model DeepAVP-TPPred was tested using stringent 5-fold cross-validation and two independent dataset testing methods, which achieved the maximum performance and showed enhanced efficiency over existing predictors in terms of both accuracy and generalization capabilities.

**Availability and implementation:**

https://github.com/MateeullahKhan/DeepAVP-TPPred.

## 1 Introduction

Viruses are severe and extensive pathogens, causing numerous infectious diseases in both humans and animals (Sébastien Calvignac-Spencer et al. 2021). The persistence of viral infections is prolonged due to variations in transmission and genetic factors (Islam and Koirala 2022). In recent times, the occurrence of zoonotic viruses such as Ebola, Zika, and SARS-CoV-2 has led to numerous chronic diseases (Phan 2020). Biologists have currently developed hundreds of antiviral medications to treat different families of viruses, including hepatitis B–C, HIV, influenza, herpes, rhinoviruses, and more (De Clercq and Li 2016). However, these antiviral medications are inadequate due to a lack of state–of–the–art novel pathogens. Furthermore, issues associated with conventional treatments include high processing time, inefficiency, and adverse side effects (Hollmann et al. 2021). In the previous decade, antiviral peptides (AVPs) have been recognized as a fundamental class of antimicrobial peptides and have been utilized in developing novel peptide-based agents for viral diseases. AVPs are short-sequence peptides derived synthetically from twenty amino acids (Gleenberg *et al.* 2007). The advantageous characteristics for developing innovative antiviral therapies include low molecular weight, minor side effects, less toxicity, and high efficiency, making them widely applicable (Ke Yan 2022).

With significant growth in genomics sequences in recent decades, computational intelligence-based peptide identification has gained the attention of biologists due to high selectivity, improved predictive results, and better generalization power. Consequently, various machine-learning approaches have been presented for predicting AVPs. Thakur *et al.* presented the pioneer computational model called AVPpred, which used alignment-based frequency representation methods to search for internal motif features from peptide sequences (Thakur *et al.* 2012). The extracted spaces were trained via a 10-fold-based support vector machine (SVM) model. Similarly, Chang *et al.* trained a random forest (RF) model by integrating aggregation, secondary structure, physiochemical properties, and computational feature encoding schemes (Chang and Yang 2013). Subsequently, AVP-IC50Pred used different machine learning models via amino acid residue composition, binary profile, and structural-based descriptors for identifying AVPs (Qureshi *et al.* 2015). Moreover, Nath *et al.* developed a stacking-based meta-model using alignment scoring and evolutionary local features of AVP samples (Nath 2021). Lissabet *et al.* presented the AntiVPP 1.0 model for predicting AVPs using an RF model using sequential residue features (Lissabet *et al.* 2019). In the PEPred-Suite model, an ensemble RF trained model is used for predicting different classes of therapeutic peptides (Wei *et al.* 2019). Additionally, a two-level feature selection was utilized to train the RF model utilizing optimal feature sets from different adaptive formulation schemes. Likewise, HybAVPnet used a two-step training strategy for identifying AVPs (Ge *et al.* 2024), where eighteen formulation methods were investigated using light-GBM and neural network-based models. Akbar *et al.* developed an ensemble training model using a genetic algorithm (Akbar *et al.* 2022). The optimal features were selected from local evolutionary features using SHAP feature selection. Similarly, the Meta-iAVP model developed another stack ensemble model using the amphiphilic-pseudo amino acid composition method. The stacking model was created by aggregating the predicted scores of GLM, RF, KNN, SVM, regression trees, and XGboost models (Schaduangrat *et al.* 2019). Pang *et al.* developed the AVPIden model by utilizing physiochemical properties, frequency, and gapped-compositional features for peptide representation (Pang *et al.* 2021). Recently, Lin *et al.* developed AI4AVP for AVPs by training deep convolutional neural networks using different types of formulation methods (Lin *et al.* 2022).

In the existing studies, we have observed that each predictor has demonstrated significant contributions to predicting AVPs. However, these models still face issues in terms of reliability and model generalization. Most existing models used sequence-based encoding methods that solely concentrated on computing the residue composition of the individual amino acids without retaining the sequence order information. Some models proposed traditional evolutionary feature descriptors, which are very time-consuming to calculate for each protein sample by searching databases. On the other hand, from a training perspective, existing models have primarily focused on traditional machine learning (ML) based trained models. Therefore, considering these issues, the existing predictors require further improvement by developing alternative solutions that can accurately discriminate between AVPs and non-AVPs with high throughput.

This work attempts to enhance the prediction performance of discriminating AVPs and non-AVPs concerning the following key aspects: (i) designing two novel feature extraction algorithms; (ii) using a new tree-based feature selection algorithm; (iii) developing a deep learning-based classification model for this study to improve the prediction performance; and (iv) showing the generalization performance with the non-experimentally non-AVPs samples. In particular, two new feature extraction algorithms, named in this study as LBP-PSSM and LBP-SMR, are proposed which are based on the image-based descriptor local binary pattern (LBP). Next, a new tree-based feature selection algorithm called the binary tree growth (BTG) algorithm is used to select the optimal feature sets from the raw heterogeneous features. A deep learning-based model deep neural network (DNN) was then designed specifically for this study as the prediction algorithm, and the performance on both benchmark training and independent datasets was tested. Finally, based on the proposed pipeline, a new forecasting algorithm for anti-viral peptides, termed DeepAVP-TPPred, is implemented. Benchmark independent testing using non-experimentally non-AVP sequences demonstrates the generalization efficacy of the model.

## 2 Materials and methods

### 2.1 Benchmark datasets

In statistical machine learning, the construction and selection of a benchmark dataset is a crucial stage for designing an intelligent predictive model. To fairly train and compare our proposed predictive model against existing state-of-the-art methods, we derived benchmark training and two independent datasets from the work of Thakur *et al.* (2012). The benchmark training dataset contains a total of 951 samples, of which 544 samples are AVPs and the remaining 407 are non-AVPs. In our study, we call this dataset as AVP951.

The first independent dataset contains 60 AVPs and 45 non-AVPs and is represented by AVP105. The second independent dataset contains 60 samples of AVPs and 60 samples of non-AVPs. However, in this study, we were unable to extract the PSSMs of 9 AVP samples. Therefore, the second dataset contains only 51 AVP samples and 60 non-AVP samples. Hence, we represent this dataset as AVP111. In AVP111, instead of experimentally verified non-AVPs, the non-experimentally non-AVPs were used to test the strength of the model.

### 2.2 Feature representation

The fact is that the majority of the predictive machine learning models handle numerical-based vectors, making it a challenging task to express a peptide sequence with numerical values or discrete models while still protecting the sequence information. The feature extraction strategy can deal with this issue. However, the use of appropriate features is one of the most crucial steps for designing highly accurate predictive models, as the success of the model purely depends on the choice of the features used when training the model. In this study, we propose new image-based feature extraction methods to represent each peptide sample as a numerical vector. The details of these feature extraction methods are as follows.

#### 2.2.1 Representation of AVP sequence as the position-specific scoring matrix

The position-specific scoring matrix is a mathematical representation of the sequence used in bioinformatics to analyze and compare biological sequences of proteins or nucleotides. Prior computational methods disclosed that evolutionary information of protein sequences is vital and widely used in a range of bioinformatics problems, such as protein DNA-binding residues (Hu *et al.* 2017), protein folding (Shen and Chou 2009), protein function prediction (Jeong *et al.* 2011), and protein secondary structure (Zangooei and Jalili 2012). Inspired by this, in the current study, we represent the AVP sequences with the PSSMs. For a given peptide sequence of length L, an L × 20 PSSM was constructed, where 20 is the number of amino acids. The following matrix $P_{\mathrm{PSSM}}$ is the general representation of the PSSM.


(1) $$P_{\mathrm{PSSM}}={\left[\begin{array}{cccc} p_{1,1} & p_{1,2} & \cdots & p_{1,20}\\ p_{2,1} & p_{2,2} & \cdots & p_{2,20}\\ \vdots & \vdots & \vdots & \vdots \\ p_{L,1} & p_{L,2} & \cdots & p_{L,20} \end{array}\right]}_{L\;\times \;20}$$

To construct the PSSMs, we used the PSI-BLAST (Schäffer *et al.* 2001) and Swiss-Prot database (Bairoch and Apweiler 1997).

##### 2.2.1.1 Transformation from PSSM to feature vector via image-based LBP method

LBP (Ojala *et al.* 1996, 2002) is a simple yet widely used feature descriptor in image analysis and computer vision for representing local features in images. Existing studies proved that LBP has a striking performance when extracting local features from the images (Huang *et al.* 2011, Shakoor 2019, Ullah *et al.* 2022). Considering the outstanding performance of LBP, we proposed an LBP-based feature extraction method for the peptide sequence. We named our proposed feature descriptor LBP-PSSM. LBP-PSSM works as follows:

First, we constructed the PSSM for each peptide sequence in our datasets and transformed its matrix representation to a PSSM image in the range of 0–255. We then choose the 3 × 3 window to calculate the LBP-PSSM features.

Next, we calculated the center pixel’s gray value of each 3 × 3 window in an image by comparing it with the neighboring pixels’ gray values using the given formula.


(2) $$\mathrm{LBP}\;-\;\mathrm{PSS}M_{N,R}=\sum_{n=0}^{N\;-\;1}s(d)2^{n}$$

where $d=p_{n}\;-\;p_{c}$ which is the difference between the center pixel $p_{c}$ and the neighborhood pixel $p_{n}$ in the N involved neighbor pixels around $p_{c}$ with the radius R. Suppose that the coordinate of $p_{c}$ is (0,0) the coordinate of $p_{n}$ are (R  cos(2πn/N),R  sin(2πn/N)). The function s(d) is used to assign a value of 1 if the intensity value of the corresponding neighborhood pixel is greater than or equal to the given threshold, and 0 otherwise. s(d) can be denoted as:


(3) $$s(d)=\{\begin{array}{l} 1,d\geq 0\\ 0,\text{otherwise} \end{array}$$

We then used the clockwise order to concatenate the obtained binary values from thresholding to form a binary pattern and finally, a histogram is generated by counting the occurrences of different patterns. In this study, a total of 256 histograms of regions were generated. These 256 histograms of regions serve as the feature vector in our research. The N=8 and R=1 were used to extract LBP-PSSM features with 256 dimensions.

#### 2.2.2 Pseudo PSSM

In this study, a 20 × 5 + 20=120-dimension of the PsePSSM feature vector is also obtained for each peptide sequence. Complete details of PsePSSM are available in Supplementary Text S1.

#### 2.2.3 Transformation from substitution matrix representation to feature vector via image-based LBP method

Substitution matrix representation (SMR), proposed by Yu *et al.* (2012), is an efficient descriptor for primary proteins. In this study, we first transform the peptide sequence to the SMR matrix. The SMR(j,l) denotes the distance of j-type amino acid contacting to the *l*^th^ position of a given peptide sequence. SMR(j,l) can be defined using the following mathematical formula:


(4) SMR(j,l)=M(j,P(l))

where M represents a 20 × 20 substitution matrix, $P=(p_{1},p_{2},p_{3},\ldots ,p_{L})$ is the given L-length peptide sequence and j=1,2,3,…,20 represents one of the twenty standard amino acid types. For the substitution matrix, we used an amino acid contact matrix (Ding *et al.* 2016). The details of the amino acid contact matrix can be found in Supplementary Table S1 under Supplementary Text S2.

The given L-length peptide sequence can then be represented by one 20 × L SMR matrix. Next, we used the same procedure discussed in Section 2.2.1.1 to transform the SMR matrix into image-based feature representation, and the resultant novel LBP-SMR feature space of 256 dimensions is obtained.

### 2.3 Binary tree growth algorithm

The extracted evolutionary and image-based heterogeneous features might uncover a range of hidden useful information that is beneficial for predicting AVPs. However, these heterogeneous features might contain irrelevant, noisy, and redundant information as well, and inputting raw heterogeneous features into a classifier may cause overfitting or underfitting. To solve this problem, a vital phase called optimized feature selection, which can extract intrinsic features from the raw heterogeneous features, is used. In this study, we also utilized the feature selection strategy by using the BTG algorithm to solve the problems mentioned above and increase the prediction efficiency. The BTG algorithm, proposed by (Too *et al.* 2018), is a powerful feature selection approach with few studies available in the literature (Kumar *et al.* 2023). The BTG algorithm is a binary version of the tree growth algorithm (Cheraghalipour *et al.* 2018). The details of the BTG algorithm are as follows:

In the first step, the initial population of trees is arbitrarily generated and then the fitness value for each tree is calculated by using the following function:


(5) $$\mathrm{Fitness}=\beta *E_{r}\;+\;(1\;-\;\beta )*\frac{\left|S\right|}{\left|F\right|}$$

where $E_{r}$ is the learning error rate, β is used to control both the prediction error and feature reduction and its value is between 0 and 1, |S| represents the number of selected features, and |F| denotes the total features in the dataset.

Next, the fitness values are used to sort out the population of trees in ascending order. The first tree group receives the best $T_{1}$ trees, and the following mathematical formula is used to generate the new tree in this group:


(6) $$N_{i}^{t\;+\;1}=\frac{N_{i}^{t}}{\theta }\;+\;rN_{i}^{t}$$

where $N_{i}$ at order i in the population denotes the tree (solution), θ denotes the trees diminution rate of power, r is the randomly disturbed number between [0,1] and t is the number of current iterations. The current tree is replaced if the newly constructed tree has a better fitness score, otherwise, it is stored for the next generation.

In the next step, $T_{2}$ trees are assigned to the second group, and for each tree, the two closest trees from the first and second groups are determined using the Euclidian distance:


(7) $$d_{i}={\left(\sum_{i=1}^{T_{1}\;+\;T_{2}}{\left(N_{T_{2}}^{t}\;-\;N_{i}^{t}\right)}^{2}\right)}^{\frac{1}{2}}$$

where $N_{T_{2}}$ denotes the present tree and $N_{i}$ represents the tree at i-th position in the population. It is worth mentioning that the distance becomes infinite when $N_{T_{2}}=N_{i}\;$, $\text{where}\;T_{2}=i$. Then, the two nearest trees $x_{1}$,$x_{2}$ with a minimum $d_{i}$ are selected and the following Equation (8) is used to compute the linear combination of the selected trees:


(8) $$Q=\lambda x_{1}\;+\;(1\;-\;\lambda )x_{2}$$

where the parameter λ is used to control the impact of the closest tree. The location of the tree in the second group is updated using:


(9) $$N_{T2}^{t\;+\;1}=N_{T2}^{t}\;+\;\alpha Q$$

where α is the angle distribution between [0,1]. The $T_{3}$ worst trees in the third group are eliminated and substituted with the new trees. Equation (10) can be used to compute the $T_{3}$:


(10) $$T_{3}=T\;-\;T_{1}\;-\;T_{2}$$

where T is the population size.

Using a masked operator, $T_{4}$ new trees are constructed within the last group around the best trees. These newly constructed trees are then added to the population and the fitness values are used to sort the merged population in ascending order. In the subsequent iteration, the best T trees are then selected to represent the new population. The process is reiterated until the termination criterion is met and finally, the universally finest tree is chosen as the best solution.

To select the optimal feature sets, the BTG algorithm utilizes a transfer function to translate the location of the trees into probability values ranging between 0 to 1. A large probability number means there will be a greater possibility that the feature will be selected. In the present study, we used the sigmoid function as the transfer function which can be expressed as:


(11) $$\delta (q_{id}^{t})=\frac{1}{1\;+\;e^{\;-q_{id}^{t}}}$$

where q denotes the *d*^th^ dimensionality of the search space. The location of the tree is updated depending on the value of the probability described below:


(12) $$q_{id}^{t\;+\;1}=\{\begin{array}{c} 1,\;\;\text{if}\;\partial <\delta_{id}^{t}\\ 0,\;\;\text{otherwise} \end{array}$$

Where ∂ is a random number between 0 and 1. The procedure of the mask operation in the BTG algorithm is shown in Table 1.

**Table 1.** A simple example showing the procedure mask operation.

New tree	1	1	0	0	0	0
Mask operator	0	1	0	1	1	0
Random finest tree	0	1	0	1	0	1
New tree after masking	1	1	0	1	0	0

In this study, we used the k-nearest neighbor (KNN) machine learning algorithm in the process of fitness evaluation because it is simple yet efficient and faster. In KNN, the value *k* is empirically set to five. Finally, a set of 352 dimensions of optimal features is obtained.

### 2.4 Prediction algorithm

**Figure 1.**
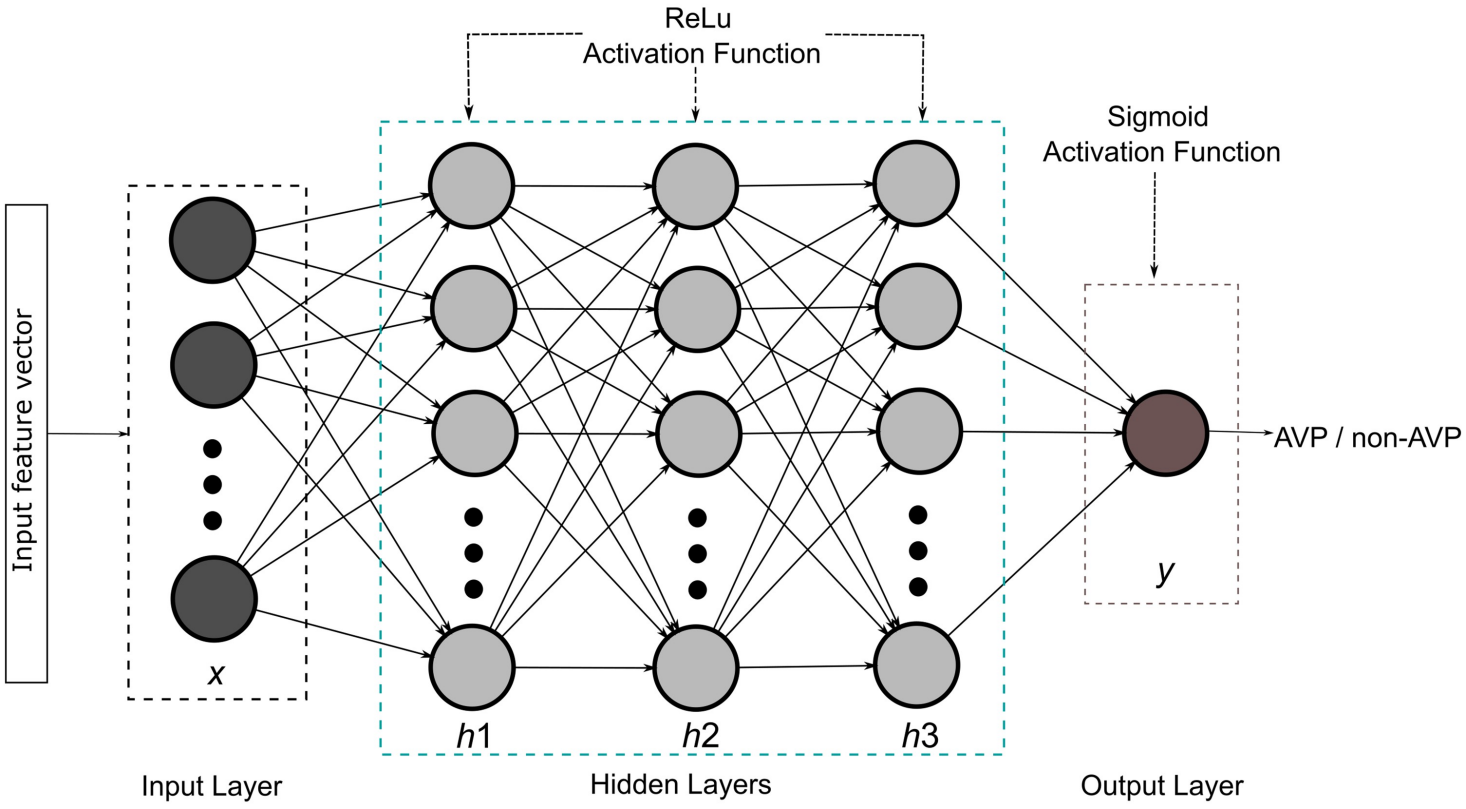
Deep neural network model architecture.

In 2006, Hinton and colleagues introduced the concept of neural networks after deriving motivation from the learning process of the human brain (Hinton *et al.* 2006, Hinton and Salakhutdinov 2006). A typical neural network incorporates an input layer, hidden layers, and an output layer. A neural network with the stacking of two or more hidden layers is referred to as a DNN. Because DNN achieves better performance than the majority of traditional machine learning models, it has been utilized in a wide area of research (Farabet *et al.* 2012, Buchan *et al.* 2023, Seo *et al.* 2023, Tsirmpas *et al.* 2024). In the present study, we trained the DNN model to construct our final prediction model for AVPs. The DNN model was trained using an input layer, three hidden layers, and an output layer. Figure 1 illustrates the DNN model designed for this study. First, the optimized hybrid features are given to the input layer (x), where each node of the input layer is associated with an instance of the input features. The input layer computes the output by using weights, a bias term, and an activation function. Next, the output of the input layer is provided as an input to the first hidden layer (h1) and using their corresponding weights, bias term, and activation function to compute the output. The process is continued until the output layer (y) is reached.

In our study, we used two activation functions rectified linear unit (ReLu), which is used at the hidden layers and sigmoid, which is used at the output layer, for predicting the input instance in the AVP or non-AVP class. The specific optimal parameters for the DNN used in this study are shown in Table 2.

**Table 2.** Optimal configuration values for the proposed DNN model.

Hyper-parameters	Optimal values
Activation function	ReLu, sigmoid
Learning rate	0.01
Number of hidden layer Neurons	128,64, 32
Optimizer	Adam
Regularization L1	0.001
Dense layers	3
Dropout rate	0.5

### 2.5 Architecture of the proposed DeepAVP-TPPred

**Figure 2.**
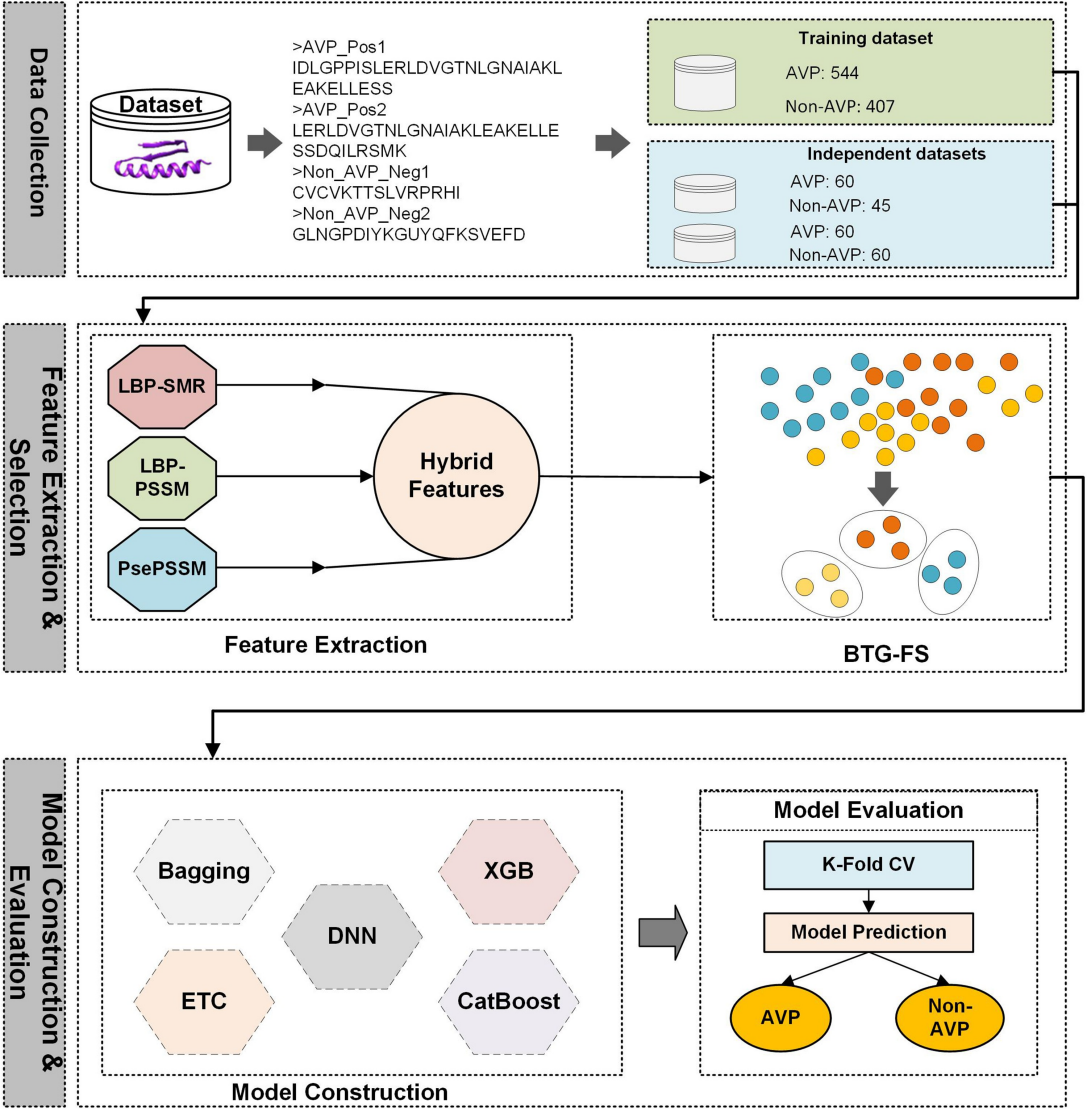
Diagrammatic overview of the proposed DeepAVP-TPPred.

Figure 2 shows a diagrammatic overview of our proposed DeepAVP-TPPred prediction algorithm. For a given peptide input sequence from the benchmark datasets, DeepAVP-TPPred first extracts the transformed PSSM-LBP, SMR-LBP, and PsePSSM feature sets by calling their respective feature description program (feature extraction phase). Next, DeepAVP-TPPred serially integrates all the extracted feature sets into a hybrid feature set and then calls the BTG feature selection algorithm to select the best optimal feature subset from the hybrid feature set (feature selection phase). Consequently, the feature set obtained from the BTG feature selection algorithm is selected as the final optimal feature set which represents the given peptide sequence. Finally, in the training phase, the obtained optimal feature set is provided to the prediction algorithm to train the prediction model (model construction). While testing the model, after producing the final optimal feature set for the given unseen peptide sequence, the trained model is called to predict the sequence as AVP or non-AVP (model evaluation phase).

### 2.6 Performance measures

In the current study, we assessed the performance of our proposed DeepAVP-TPPred using various performance assessment measures, i.e. Accuracy (Acc), Sensitivity (Sen), Specificity (Sp), and the Matthew correlation coefficient (MCC). The mathematical notations for Acc, Sen, Sp, and MCC are provided in Supplementary Equations (6)–(9) under Supplementary Text S3. In addition to that, we also assessed the model performance on a broad level by computing the area under the receiver operating characteristic (ROC) curve (AUC) and the area under the precision-recall (AUPR) curve, which are other critical assessment tools.

### 2.7 Model evaluation

In machine learning, various model evaluation strategies are used to test the performance of a prediction model, such as the *k*-fold cross-validation (CV), jackknife, and independent testing strategies. The major limitations of the jackknife test are the computational time and the huge number of calculations. Therefore, in this study, we utilized the *k*-fold CV method to avoid overfitting and boost the generalization capability of the model. *k*-fold CV method randomly divides the training dataset into *k* non-overlapping approximately equal-sized subsets and at each step, the model is trained on *k*-1 subsets and tested on the left-out subset. In this work, we used the value of k=5.

An Independent test is the most critical testing method for assessing the generalization ability of a model. Therefore, in this study, we also used the independent testing method by using two unseen datasets to show the efficacy of the proposed model.

## 3 Results

### 3.1 Performance analysis of individual feature sets using various learning models on the training dataset

**Figure 3.**
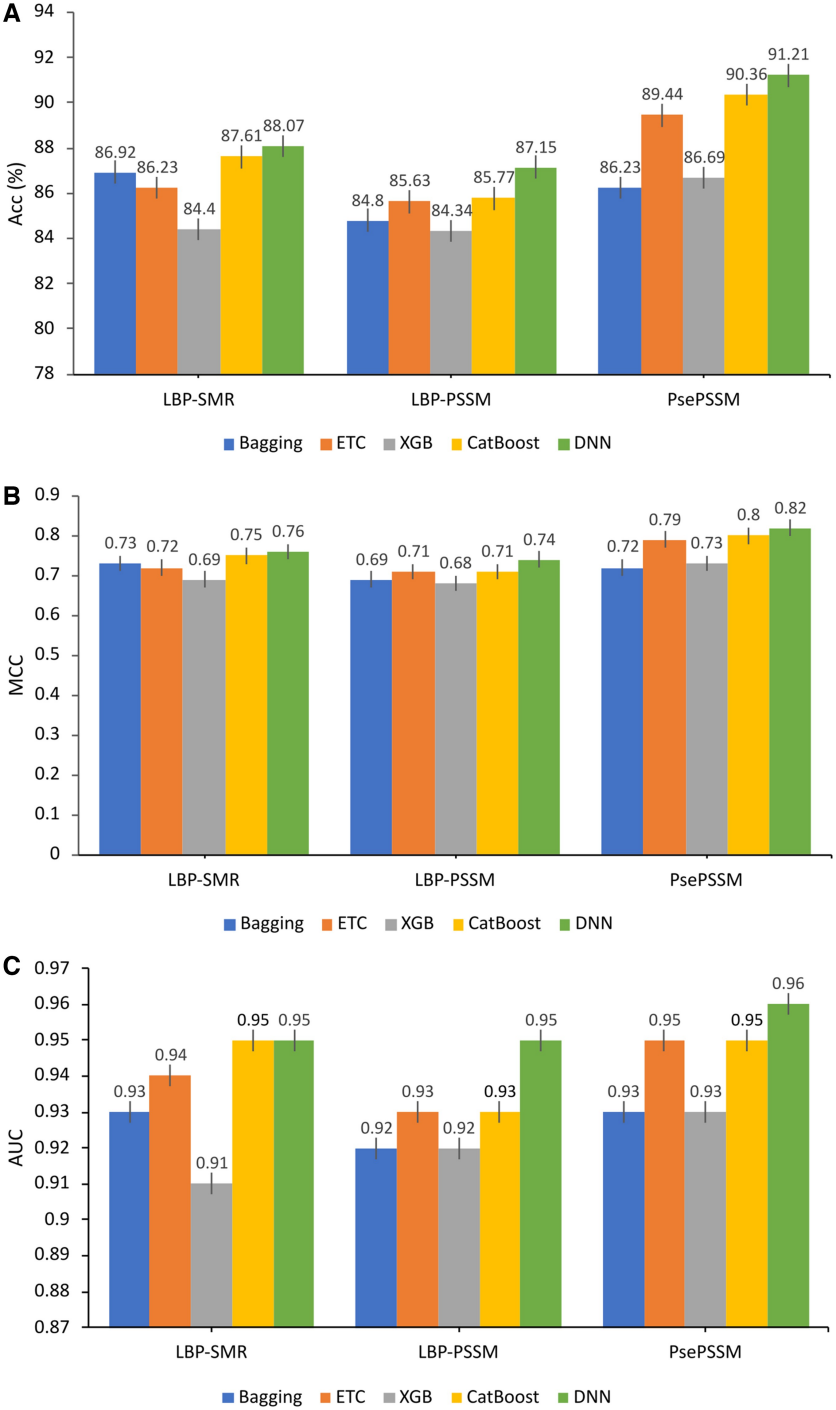
Performance comparison of different classifiers on the individual feature sets: (A), (B), and (C) show the comparison in terms of Acc, MCC, and AUC values, respectively.

To show the performance of the proposed feature extraction algorithms, we tested using various latest classification learning models, including Bagging, Extra-Trees Classifier (ETC), eXtreme Gradient Boosting (XGB), CatBoost, and DNN, using 5-fold cross-validation on the benchmark training dataset. The hyper-parameter settings for the Bagging, ETC, XGB, and CatBoost are provided in Supplementary Table S2 under Supplementary Text S4. The comparative results are shown in Table 3 and Fig. 3 is provided to help easily understand the effect of different classifiers. From Table 3, we can see that both the LBP-SMR and LBP-PSSM individual raw feature sets have more reasonable and competitive performance on all the classifiers. Between the two, the LBP-SMR achieved the best performance on the DNN classifier with an Acc and MCC of 88.07 and 0.76, respectively. Similarly, the LBP-PSSM achieved Acc = 87.15 and MCC = 0.74. Both the LBP-SMR and LBP-PSSM achieved an equal AUC of 0.95 on the DNN classifier, which shows the effectiveness of our proposed feature extraction methods.

**Table 3.** Prediction analysis of individual feature sets using training samples.

Method	Classifier	Acc (%)	Sen (%)	Sp (%)	MCC	AUC
LBP-SMR	Bagging	86.92	83.97	89.87	0.73	0.93
	ETC	86.23	90.90	95.93	0.72	0.94
	XGB	84.40	87.87	91.79	0.69	0.91
	CatBoost	87.61	87.39	87.87	0.75	0.95
	DNN	88.07	84.03	92.91	0.76	0.95
LBP-PSSM	Bagging	84.80	84.34	85.26	0.69	0.92
	ETC	85.63	85.26	86.03	0.71	0.93
	XGB	84.34	82.87	85.81	0.68	0.92
	CatBoost	85.77	85.71	85.84	0.71	0.93
	DNN	87.15	84.03	90.91	0.74	0.95
PsePSSM	Bagging	86.23	89.07	82.81	0.72	0.93
	ETC	89.44	84.03	95.91	0.79	0.95
	XGB	86.69	87.39	85.85	0.73	0.93
	CatBoost	90.36	89.91	90.91	0.80	0.95
	DNN	91.21	89.46	92.97	0.82	0.96

**Figure 4.**
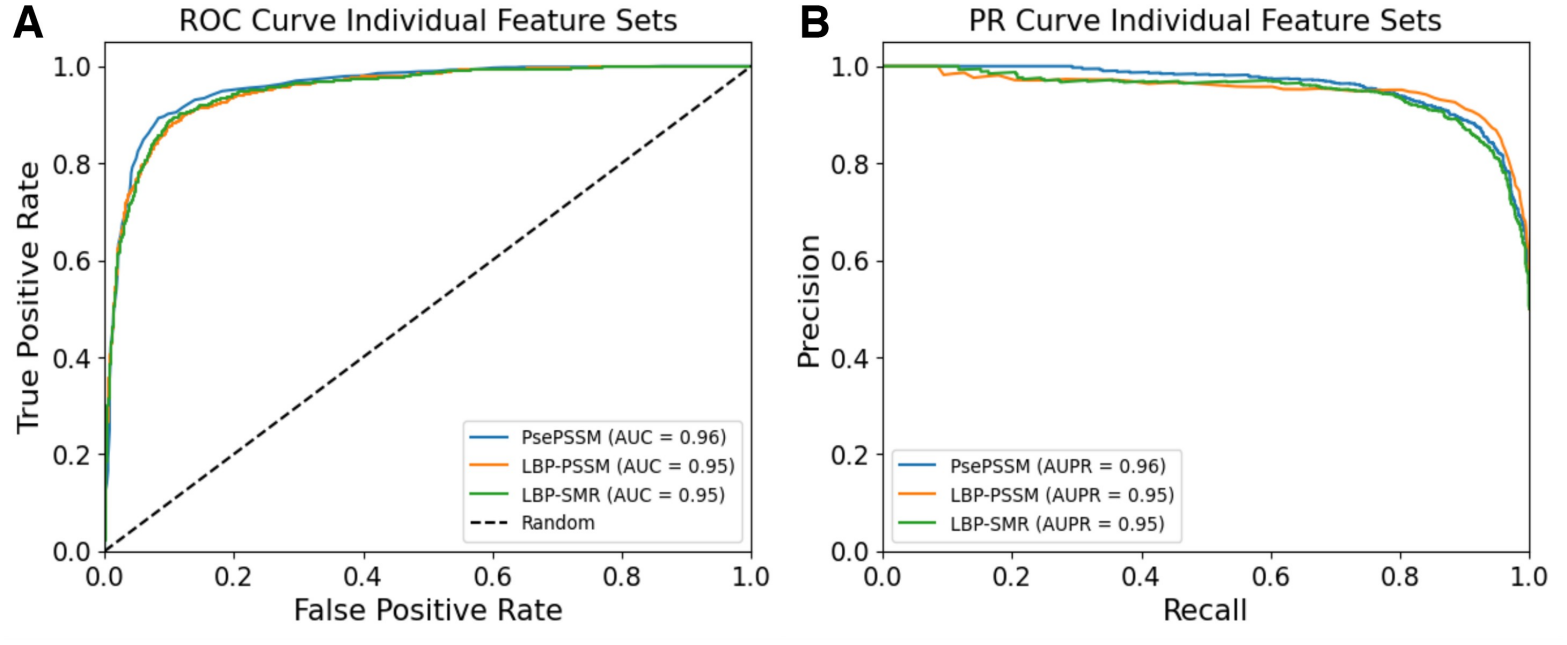
Performance comparison of individual features using DNN classifier: (A) shows the comparison in terms of ROC and; (B) shows the comparison in terms of PR curves.

Similarly, by comparing the experimental results of all the classifiers in Fig. 3, The DNN classifier performed better by achieving the Acc, MCC, and AUC of 91.21, 0.82, and 0.96 on the PsePSSM, 88.07, 0.76, and 0.95 on the LBP-SMR and 87.15, 0.74 and 0.95 on the LBP-PSSM, respectively, which shows that DNN is more powerful than the rest of the classifier when using individual raw feature sets. The CatBoost classifier achieved the second-best performance in comparison to other classifier results, while the remaining classifiers have more competitive performance results. From Table 3, we can easily conclude that both the proposed feature extraction methods achieved better performance because the LBP-SMR and LBP-PSSM capture more intrinsic information. Similarly, from the comparison in Fig. 3, we observed that the DNN performs better than even the updated classifiers, which shows its superiority. Therefore, we further performed experiments, in terms of ROC and PR curves, using the DNN classifier and the results were illustrated in Fig. 4A and B, respectively.

Figure 4A shows the ROC curves, while Fig. 4B shows the PR curves for the individual features. From both Fig. 4A and B, we can see that the proposed features have more stable performance in terms of both AUC and AUPR values, which again demonstrates the effectiveness of the LBP-PSSM and LBP-SMR feature extraction methods.

### 3.2 BTG improves the model performance

Next, to further improve the performance of the DeepAVP-TPPred model, we integrated all three extracted feature sets in serial fashion and named it as hybrid features, i.e. Hybrid Features = LBP-SMR+LBP-PSSM+PsePSSM. The results of the hybrid features on different classifiers, using a 5-fold CV and benchmark training dataset, are provided in Table 4. By comparing the results in Tables 3 and 4, we observed that by integrating all three feature sets, the model accuracy for most of the classifiers improved drastically. Notably, by comparing the results of the best individual feature set PsePSSM, which achieved the Acc, MCC, and AUC of 91.21, 0.82, and 0.96, respectively, on the DNN classifier using the benchmark training dataset, and Hybrid Features, which achieved the Acc = 95.39, MCC = 0.90, and AUC = 0.97, the Acc, MCC and AUC of the hybrid features increased by 4.18%, 8%, and 1%, respectively. This performance increase is because each feature set contains unique information and integrating all the unique information will result in better performance. However, there may also be redundant, noisy, and irrelevant information in the integrated feature set, which might not convey the maximum performance. Therefore, we passed the hybrid feature set through the BTG feature selection algorithm to remove noisy, irrelevant, and redundant information. The resultant feature set is called the optimal feature set, which is denoted here as hybrid features+BTG.

**Table 4.** Prediction outcomes of hybrid features before and after BTG feature selection algorithm using 5-fold CV on the benchmark training dataset.

Method	Classifier	Acc (%)	Sen (%)	Sp (%)	MCC	AUC
Hybrid features	Bagging	89.90	87.39	92.91	0.79	0.95
	ETC	91.28	90.75	91.94	0.82	0.95
	XGB	89.99	87.81	92.12	0.80	0.95
	CatBoost	91.28	87.39	95.96	0.83	0.94
	DNN	95.39	95.48	95.16	0.90	0.97
Hybrid features + BTG	Bagging	91.28	90.76	91.92	0.82	0.95
	ETC	91.74	89.91	93.94	0.83	0.96
	XGB	92.20	90.75	93.94	0.84	0.96
	CatBoost	92.75	95.51	90.14	0.85	0.96
	DNN	96.84	96.92	98.66	0.93	0.98

The result of the hybrid features+BTG is also provided in Table 4. We can easily conclude from Table 4 that in terms of all performance measures, the hybrid features+BTG achieved increased performance on all the classifiers with DNN at the front in comparison to the simple hybrid features or any individual feature set, which shows that BTG feature selection can further empower the prediction performance of the DeepAVP-TPPred using 5-fold CV on the benchmark training dataset.

**Figure 5.**
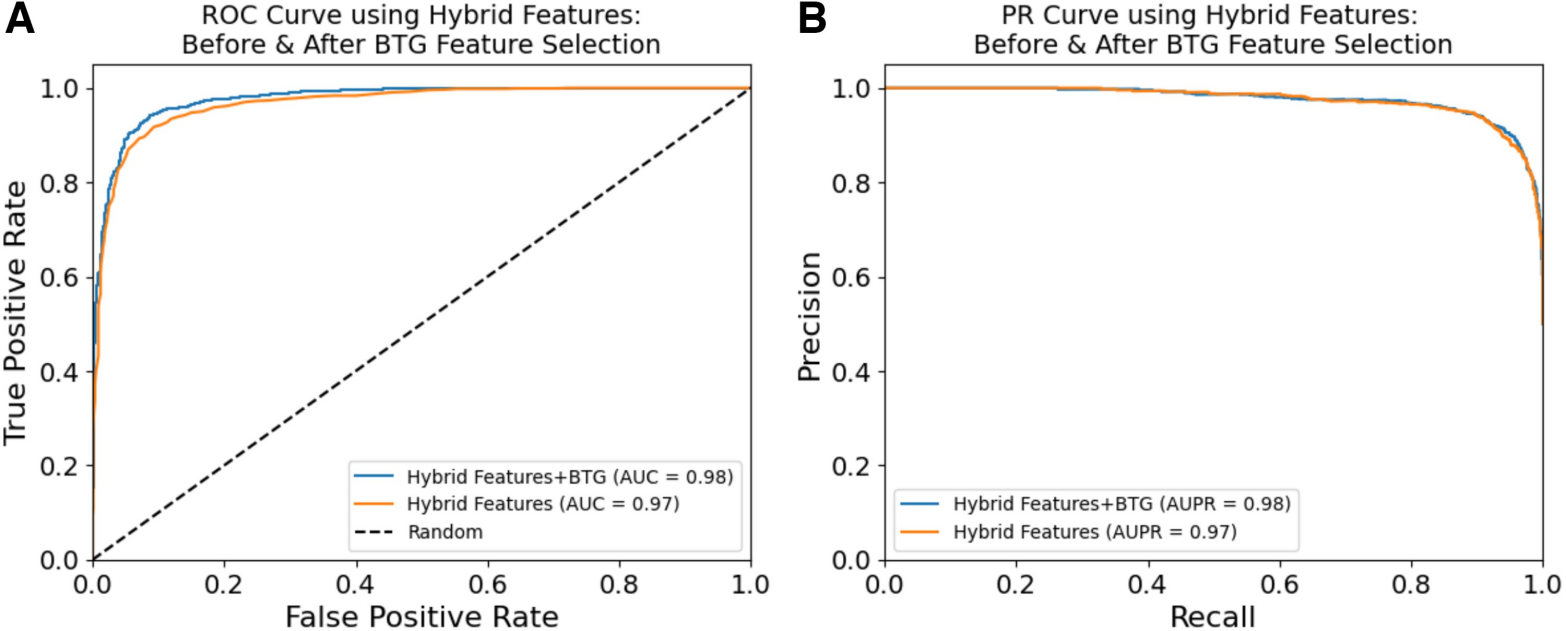
Performance comparison of Hybrid features before and after BTG feature selection: (A) shows the comparison in terms of ROC and; (B) shows the comparison in terms of PR curves for the DNN classifier.

To further validate the results on a wider level, we compared both the Hybrid features before and after applying the BTG feature selection algorithm. The results are illustrated in Fig. 5. From Fig. 5A, which shows the ROC curves, and Fig. 5B, which shows the PR curves for Hybrid features and Hybrid Fetures+BTG, respectively, it can be concluded that applying BTG on the Hybrid Features, we can see improvement in both AUC and AUPR values on the obtained Hybrid Fetures+BTG optimal feature set. Furthermore, to illustrate the high contributory features, we also performed SHapley Additive exPlanations (SHAP) analysis-based interpolation as shown in Supplementary Fig. S1 in Supplementary Text S5. In addition, we also performed heat map analysis of the encoding schemes versus trained classifiers using predicted Acc and MCC as shown in Supplementary Fig. S2A and B under Supplementary Text S5.

### 3.3 DeepAVP-TPPred has enhanced generalized performance

In the previous Results and Discussion sections, we have shown results by performing experiments on the training dataset using a 5-fold CV. To validate the assumption that training our DeepAVP-TPPred with DNN as a learning model has enhanced performance, in this section, we performed an independent testing method using two different types of independent datasets, the details of which are discussed in Section 2.1, and compared the results with other classifiers. The results derived from the independent testing experiments for all the classifiers are shown in Table 5. We can see from the results that both the independent datasets AVP105 and AVP111 have better performance outcomes in terms of generalization capability. Specifically, when the DNN is used, the DeepAVP-TPPred has the maximum Acc and MCC of 96.09 and 0.92 on the AVP105 and 95.73 and 0.92 on the AVP111 in comparison with the second-best classifier CatBoost results, which are 81.44 and 0.62 on the AVP105 and 79.16 and 0.60 on the AVP111, respectively, in Table 5. Similarly, in terms of Sen, Sp, and AUC, the DNN achieved better performance than all the other classifiers, which again demonstrates that the DeepAVP-TPPred with the DNN classifier has better generalization capability.

**Table 5.** Prediction outcomes using independent datasets.

Dataset	Method	Classifier	Acc (%)	Sen (%)	Sp (%)	MCC	AUC
AVP105	Hybrid features + BTG	Bagging	76.26	74.57	77.96	0.52	0.91
		ETC	78.07	76.27	79.66	0.55	0.90
		XGB	71.15	79.66	62.71	0.42	0.78
		CatBoost	81.44	79.66	83.05	0.62	0.89
		DNN	96.09	94.83	97.33	0.92	0.98
AVP111	Hybrid features + BTG	Bagging	76.16	85.37	66.73	0.53	0.81
		ETC	75.00	66.67	83.31	0.50	0.82
		XGB	70.83	58.83	83.33	0.43	0.78
		CatBoost	79.16	66.67	91.67	0.60	0.85
		DNN	95.73	95.38	97.77	0.92	0.97

**Figure 6.**
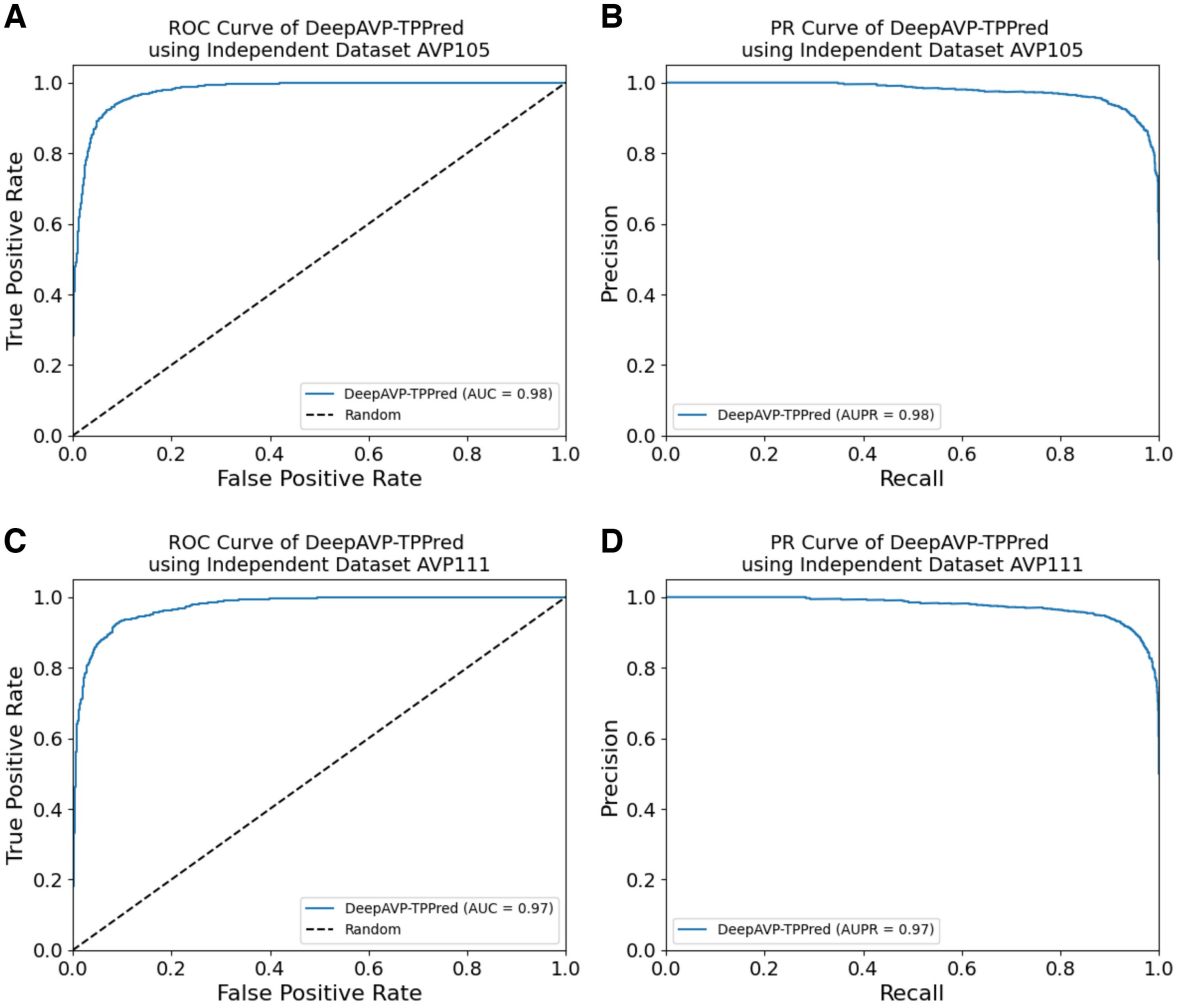
Performance evaluation of DeepAVP-TPPred using independent datasets: Panels (A) and (B) show the ROC and PR curve for the AVP105 dataset while panels (C) and (D) show the ROC and PR curves for the AVP111 dataset.

We further evaluated the performance of the DeepAVP-TPPred in terms of ROC and PR curves and the results are provided in Fig. 6. From Fig. 6A and D, which show the ROC curves on the AVP105 and AVP111 independent datasets, we can observe that the DeepAVP-TPPred has a smoother and more consistent performance in terms of AUC values. Similarly, in terms of PR curves shown in Fig. 6B and D on the AVP105 and AVP111 independent datasets, respectively, the AUPR values once again evaluate the effectiveness of the DeepAVP-TPPred in terms of generalization capability.

### 3.4 Performance comparison of DeepAVP-TPPred and other existing methods on the AVP951 training dataset

In this section, in order to illustrate the predictive performance of our proposed DeepAVP-TPPred, we compared it with other existing binary class AVPs prediction methods, including AVPpred (Thakur *et al.* 2012), Meta-iAVP (Schaduangrat *et al.* 2019), and Chang *et al.*’s method (Chang and Yang 2013). The performance results of DeepAVP-TPPred against these predictors, in terms of ACC, Sen, Sp, and MCC, are shown in Table 6. The results of other methods are derived from their respective papers.

**Table 6.** Performance comparison of DeepAVP-TPPred with existing models using training dataset.

Method	Acc (%)	Sen (%)	Sp (%)	MCC
AVPpred	85.00	82.20	88.20	0.70
Chang *et al.*	85.10	86.60	83.00	0.70
Meta-iAVP	88.20	89.20	86.90	0.76
DeepAVP-TPPred	96.84	96.92	98.66	0.93

We can see from Table 6 that the proposed DeepAVP-TPPred substantially enhanced all the performance measures. More specifically, DeepAVP-TPPred achieved Acc = 96.84 and MCC = 0.93, which were 11.84% and 23%, 11.74% and 23%, 8.64% and 17%, respectively, higher than the AVPpred, Chang *et al.* method and Meta-iAVP method. Similarly, in terms of Sen and Sp, our DeepAVP-TPPred also has better performance than the other existing methods in Table 6. Altogether, the performance results on the training dataset suggest that DeepAVP-TPPred outperformed the other existing sequence-based AVP methods.

### 3.5 Performance comparison of DeepAVP-TPPred with existing methods on the independent datasets

**Figure 7.**
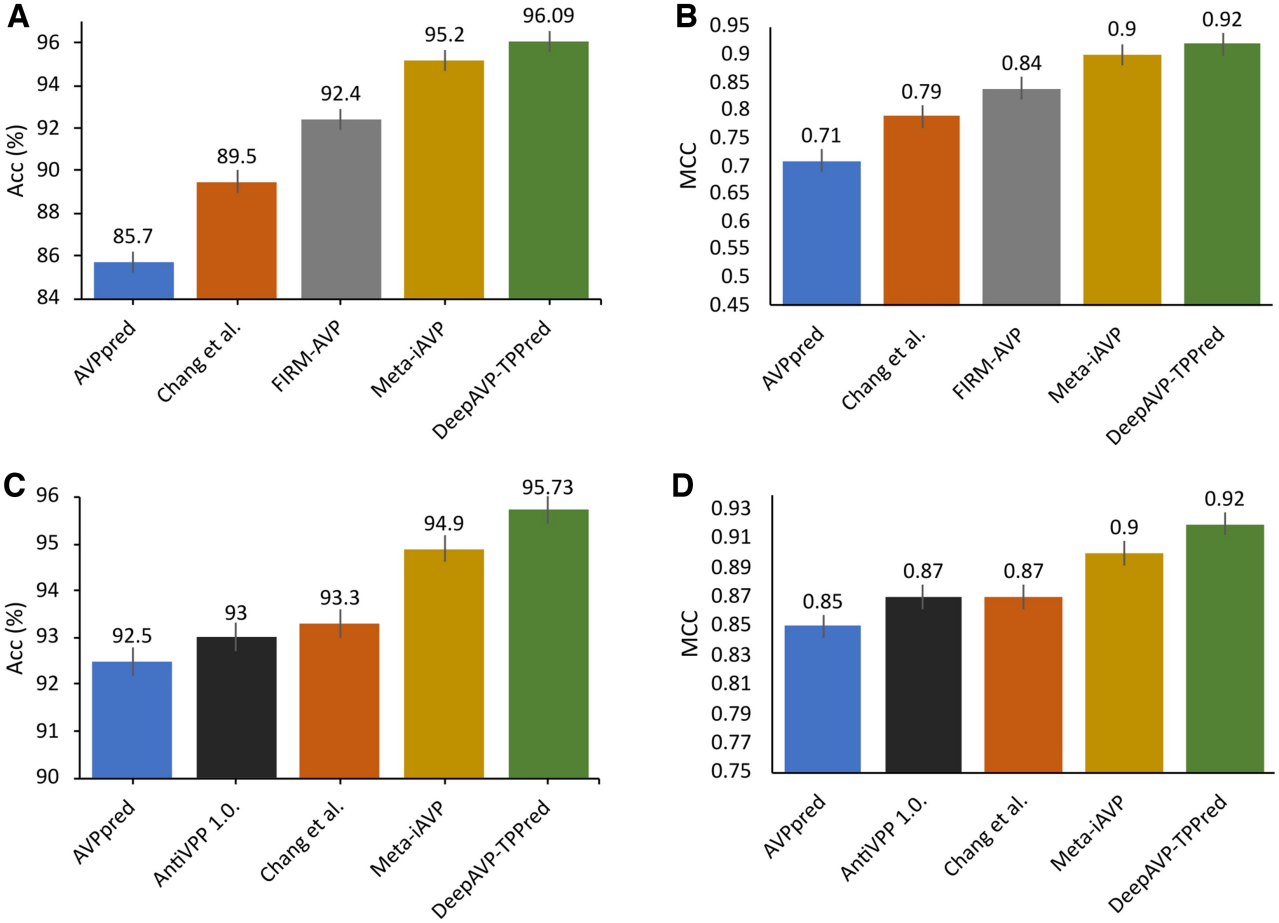
Performance comparison of DeepAVP-TPPred with existing models using independent datasets: Panels (A) and (C) show the Acc and panels (B) and (D) show the MCC on AVP105 and AVP111, respectively.

Figure 7 further illustrates the generalization power of the proposed DeepAVP-TPPred and other existing sequence-based AVP methods on the two independent datasets AVP105 and AVP111, including AVPpred, Chang *et al.*’s method, FIRM-AVP (Chowdhury *et al.* 2020), AntiVPP1.0 (Lissabet *et al.* 2019), and Meta-iAVP. Again, the performance results of these methods are derived from their respective papers.

We can observe from Fig. 7A and D that DeepAVP-TPPred achieved the best predictive performance with Acc = 96.09 and MCC = 0.92 on the AVP105, Acc = 95.73 and MCC = 0.92 on the AVP111, which were about 0.89–10.39% and 2–21%, 0.83–3.23%, and 2–7% higher than the existing methods. More specifically, in terms of Acc and MCC, DeepAVP-TPPred is 0.89% and 2%, 0.83%, and 2% higher than the runner-up Meta-iAVP, 10.39% and 3.23%, 21%, and 7% higher than the AVPpred, 6.59% and 2.43%, 13% and 5% higher than Chang *et al.*’s method, on the AVP105 and AVP111 datasets, respectively. Similarly, DeepAVP-TPPred is 3.69% and 8% higher than the FIRM-AVP on the AVP105, while 2.73% and 5% higher than the AntiAVP 1.0 on the AVP111 in terms of Acc and MCC, respectively. The comparative results of all these methods in terms of additional performance measures are provided in Supplementary Table S3 in Supplementary Text S6, which also shows that DeepAVP-TPPred substantially improved those other performance measures. On the other hand, local interpretable model-agnostic explanations (LIME) analysis was also performed to validate the proposed model more effectively, as shown in Supplementary Fig. S3 under Supplementary Text S6. Together, all the results in Fig. 7A and D, Supplementary Table S3 and Supplementary Fig. S3 show that the proposed DeepAVP-TPPred has better generalization capability than the other existing methods.

## 4 Discussion

Accurate forecasting of AVPs is critically essential for developing novel peptide-based agents for viral diseases. To boost the performance of the AVP’s prediction, this study proposed a computational-based predictor, namely DeepAVP-TPPred. Precisely, DeepAVP-TPPred first extracted various features from each peptide sequence by using PsePSSM and two proposed LBP-PSSM and LBP-SMR feature extraction methods. Next, the extracted feature sets are integrated in a serial way to generate a more powerful heterogeneous feature set to compensate for the limitations of single feature sets. Using a new tree-based feature selection algorithm, i.e. BTG, DeepAVP-TPPred then selected the optimal hybrid feature set from the integrated feature set, which further outputted an effective feature vector. The designed DNN model was then learned on the optimal feature sets to construct the DeepAVP-TPPred model. Benchmark experiments on the AVP training and independent datasets demonstrated the superiority of DeepAVP-TPPred over several existing state-of-the-art binary class prediction models for AVPs. The remarkable performance of the DeepAVP-TPPred is mainly due to the capturing of more meaningful information in the PSSM and SMR of each sequence by LBP-PSSM and LBP-SMR, which can extract more local evolutionary information, and utilizing the tree-based BTG feature selection algorithm which can further generate a more efficient optimal feature set. The careful selection of the learning model further resulted in better predictive performance.

In the future, we plan to deal with the following different aspects and further enhance the prediction performance of DeepAVP-TPPred: (i) constructing larger AVPs datasets; (ii) extracting deep learned-based features and integrating with other effective feature sets, such as features from residue energy contact matrix representation (RECM), to construct a more robust feature set; (iii) integrating feature extraction algorithm in ways other than simple serial integration approach; (iv) designing more advanced learning models, for instance, the ensemble of deep learned models; and (v) developing and designing a user-friendly web server.

## Supplementary Material

btae305_Supplementary_Data
