# Supplementary material for: DeepAVP-TPPred: identification of antiviral peptides using transformed image-based localized descriptors and binary tree growth algorithm
Source: Bioinformatics. 2024 May 6;40(5):btae305. doi: 10.1093/bioinformatics/btae305 (PMC11256913; doi:10.1093/bioinformatics/btae305)
Supplement: btae305_Supplementary_Data [file btae305_supplementary_data.docx]

DeepAVP-TPPred: identification of antiviral peptides using transformed image-based localized descriptors and binary tree growth algorithm

Matee Ullah^1^, Shahid Akbar^1,2^, Ali Raza^3^, and Quan Zou^1,4,*^

^1^Institute of Fundamental and Frontier Sciences, University of Electronic Science and Technology of China, Chengdu 610054, Sichuan, China. ^2^Department of Computer Science and Technology, Abdul Wali Khan University Mardan, Mardan 23200, Pakistan. ^3^Department of Computer Science, MY University, Islamabad 45750, Pakistan. ^4^Yangtze Deta Region Institute (Quzhou), University of Electronic Science and Technology of China, Quzhou, 324003, Zhejiang, China.

*To whom correspondence should be addressed.

# **Text S1. PsePSSM**

PSSM contains evolutionary information but it overlooks the sequence order information. An extended version of the simple PSSM, called Pseudo position-specific scoring matrix, is used to attain the other relevant information from the peptide sequence (Chou and Shen, 2007). PsePSSM has been applied to a range of different tasks (Ding, et al., 2020; Lim, et al., 2019; Qiu, et al., 2018; Wang, et al., 2020). The following series of steps are utilized to calculate the PsePSSM features of the peptide sequence.

First, the PSSM representation for the corresponding peptide sequence is obtained as discussed in section 2.2.1. Next, we compute the PSSM composition which is a vector of 20 elements. Let $P_{PSSM}$ be the PSSM for a given peptide sequence $P$, the PSSM composition can be defined as

$$\bar{P}_{PSSM}=[\bar{p}_{1},\bar{p}_{2},\bar{p}_{3},...,\bar{p}_{20}]^{T}$$

where $\bar{p}_{j}$ is a vector element in $\bar{P}_{PSSM}$ and can be obtained using:

$$\bar{p}_{j}\frac{1}{L}\sum_{i=1}^{L} p_{i,j}\text{ (1}\leq j\leq\text{20)}$$

Where $p_{i,j}$ represents the $j^{th}$ residue at position $i$ in the $P_{PSSM}$. After obtaining the PSSM composition $\bar{P}_{PSSM}$ which lacks the sequence order information, we calculate the $d\text{-tier}$ correlation factor $\lambda$ for each column of the $P_{PSSM}$ using:

$$\lambda^{d}=\left[\lambda_{1}^{d},\text{ }\lambda_{2}^{d}\text{ },\lambda_{3}^{d}\text{ },...,\text{ }\lambda_{20}^{d}\right]$$

where

$$\lambda_{j}^{d}=\frac{1}{L-d}\sum_{i=1}^{L-d} (p_{i,j}-p_{i+d,j})^{2},\text{ (}1\leq j\leq20)$$

$\lambda_{j}^{d}$ denotes the correlation factor of the $P_{PSSM}$ score of the two amino acids separated by $d$ $(1\leq d\leq D)$, $(D\leq L)$. Finally, the PsePSSM $f_{PsePSSM}$ can be obtained by combining the PSSM composition $\bar{P}_{PSSM}$ and correlation factor $\lambda^{d}$ as follows:

$$f_{PsePSSM}=\left[\bar{p}_{1},\bar{p}_{1},...,\bar{p}_{1},\lambda_{1}^{d},\lambda_{2}^{d},\lambda_{3}^{d},...,\lambda_{20}^{d}\right]^{T}$$

As there is no theoretical justification for choosing the optimal value of $\lambda$ (Arif, et al., 2022), we empirically applied the grid-search approach between 1 to 9 with an interval of 1 and the optimal value $\lambda=5$, is selected using the training dataset (AVP951).

# **Text S2. Amino Acid Contact Matrix**

The following Table S1 is the amino acid contact matrix used as a substitution matrix in this study. The amino acid contact matrix is a $20\times20$ matrix which is obtained from the statistically observed correlated frequencies of pairwise contacts on the interface.

| **Table S1.** The Amino Acid Contact Matrix used in this study. | -1.32 | -0.57 | -0.66 | -0.65 | -0.68 | -0.46 | -0.59 | -1.14 | -0.84 | -0.92 | -0.95 | -0.47 | -0.93 | -0.87 | -0.61 | -0.89 | -0.68 | -0.66 | -0.78 | -0.94 |
| --- | --- | --- | --- | --- | --- | --- | --- | --- | --- | --- | --- | --- | --- | --- | --- | --- | --- | --- | --- | --- |
|  | -0.57 | -0.77 | -0.48 | -1.04 | -0.22 | -1.07 | -0.6 | -0.68 | -0.48 | -0.49 | -0.48 | -0.43 | -0.53 | -0.4 | -0.5 | -0.58 | -0.57 | -0.46 | -0.54 | -0.54 |
|  | -0.66 | -0.48 | -1.34 | -0.52 | -0.51 | -0.65 | -0.76 | -0.75 | -0.81 | -0.62 | -0.49 | -0.54 | -0.64 | -0.48 | -0.88 | -0.74 | -0.9 | -0.31 | -0.57 | -0.53 |
|  | -0.65 | -1.04 | -0.52 | -0.87 | -0.39 | -0.45 | -0.67 | -0.54 | -0.72 | -0.35 | -0.28 | -1.12 | -0.64 | -0.24 | -0.52 | -0.57 | -0.63 | -0.51 | -0.51 | -0.43 |
|  | -0.68 | -0.22 | -0.51 | -0.39 | -2.41 | -0.12 | -0.68 | -1.1 | -0.87 | -0.93 | -0.93 | -0.25 | -1.05 | -0.72 | -0.6 | -0.87 | -0.63 | -0.75 | -0.65 | -1.05 |
|  | -0.46 | -1.07 | -0.65 | -0.45 | -0.12 | -0.97 | -0.51 | -0.59 | -0.69 | -0.41 | -0.4 | -1.24 | -0.39 | -0.42 | -0.68 | -0.65 | -0.39 | -0.34 | -0.48 | -0.58 |
|  | -0.59 | -0.6 | -0.76 | -0.67 | -0.68 | -0.51 | -1.07 | -0.85 | -0.57 | -0.44 | -0.68 | -0.61 | -0.61 | -0.64 | -0.62 | -0.78 | -0.77 | -0.69 | -0.63 | -0.58 |
|  | -1.14 | -0.68 | -0.75 | -0.54 | -1.1 | -0.59 | -0.85 | -1.77 | -0.91 | -0.95 | -0.73 | -0.5 | -0.84 | -0.9 | -1.07 | -0.71 | -1.05 | -0.81 | -0.78 | -0.8 |
|  | -0.84 | -0.48 | -0.81 | -0.72 | -0.87 | -0.69 | -0.57 | -0.91 | -1.09 | -0.53 | -0.49 | -0.34 | -0.52 | -0.56 | -0.53 | -0.6 | -0.79 | -0.6 | -0.65 | -0.7 |
|  | -0.92 | -0.49 | -0.62 | -0.35 | -0.93 | -0.41 | -0.44 | -0.95 | -0.53 | -1.34 | -0.99 | -0.38 | -1 | -0.91 | -0.63 | -0.57 | -0.66 | -0.59 | -0.58 | -0.93 |
|  | -0.95 | -0.48 | -0.49 | -0.28 | -0.93 | -0.4 | -0.68 | -0.73 | -0.49 | -0.99 | -1.22 | -0.33 | -0.88 | -0.91 | -0.71 | -0.64 | -0.85 | -0.75 | -0.53 | -0.93 |
|  | -0.47 | -0.43 | -0.54 | -1.12 | -0.25 | -1.24 | -0.61 | -0.5 | -0.34 | -0.38 | -0.33 | -0.91 | -0.49 | -0.3 | -0.33 | -0.6 | -0.6 | -0.42 | -0.51 | -0.36 |
|  | -0.93 | -0.53 | -0.64 | -0.64 | -1.05 | -0.39 | -0.61 | -0.84 | -0.52 | -1 | -0.88 | -0.49 | -1.66 | -0.86 | -0.94 | -0.65 | -0.58 | -0.88 | -0.52 | -1 |
|  | -0.87 | -0.4 | -0.48 | -0.24 | -0.72 | -0.42 | -0.64 | -0.9 | -0.56 | -0.91 | -0.91 | -0.3 | -0.86 | -1.17 | -0.5 | -0.68 | -0.64 | -0.84 | -0.67 | -0.85 |
|  | -0.61 | -0.5 | -0.88 | -0.52 | -0.6 | -0.68 | -0.62 | -1.07 | -0.53 | -0.63 | -0.71 | -0.33 | -0.94 | -0.5 | -1.62 | -0.9 | -0.54 | -0.97 | -0.93 | -0.77 |
|  | -0.89 | -0.58 | -0.74 | -0.57 | -0.87 | -0.65 | -0.78 | -0.71 | -0.6 | -0.57 | -0.64 | -0.6 | -0.65 | -0.68 | -0.9 | -1.27 | -0.85 | -0.28 | -0.67 | -0.68 |
|  | -0.68 | -0.57 | -0.9 | -0.63 | -0.63 | -0.39 | -0.77 | -1.05 | -0.79 | -0.66 | -0.85 | -0.6 | -0.58 | -0.64 | -0.54 | -0.85 | -1.19 | -0.7 | -0.57 | -0.68 |
|  | -0.66 | -0.46 | -0.31 | -0.51 | -0.75 | -0.34 | -0.69 | -0.81 | -0.6 | -0.59 | -0.75 | -0.42 | -0.88 | -0.84 | -0.97 | -0.28 | -0.7 | -1.15 | -0.64 | -0.64 |
|  | -0.78 | -0.54 | -0.57 | -0.51 | -0.65 | -0.48 | -0.63 | -0.78 | -0.65 | -0.58 | -0.53 | -0.51 | -0.52 | -0.67 | -0.93 | -0.67 | -0.57 | -0.64 | -0.82 | -0.58 |
|  | -0.94 | -0.54 | -0.53 | -0.43 | -1.05 | -0.58 | -0.58 | -0.8 | -0.7 | -0.93 | -0.93 | -0.36 | -1 | -0.85 | -0.77 | -0.68 | -0.68 | -0.64 | -0.58 | -1.23 |

# **Text S3. Mathematical Notations for Performance measures**

Following are the mathematical notations for the Accuracy (Acc), Sensitivity (Sen), Specificity (Sp) and the Matthew correlation coefficient (MCC).

where $TP$ represents true AVPs that are correctly predicted by the model as positive instances, $TN$ represents true non-AVPs that are correctly classified by the model as negative instances, $FN$ represents AVPs that are mistakenly predicted as non-AVPs and $FP$ represents non-AVPs that are mistakenly predicted as AVPs.

# **Text S4. Performance comparison with existing methods on the independent datasets**

The Optimal Hyper Parameters for CatBoost, XGB, ETC and Bagging classifiers are provided in the following Table S2.

**Table S2.** Hyper Parameters for Classifiers Learning Models

| **Methods** | **Parameter** | **Optimal Value** |
| --- | --- | --- |
| CatBoost | Iteration | 5 |
|  | Learning_rate | 0.1 |
|  | Depth | 6 |
|  | L2_leaf_reg | 3.0 |
|  | Border_count | 16 |
|  | Loss_function | Logloss |
|  | Early_stopping_rounds | 10 |
|  | Random_seed | 42 |
| XGB | No. of estimators | 200 |
|  | Learning rate | 0.001 |
|  | max depth | 15 |
|  | reg_lambda | 2 |
|  | objective function | binary-logistic |
|  | gamma | 1 |
|  | booster | gbtree |
|  | reg_alpha | 1 |
| ETC | Random_state  N_etimator | 42 |
|  |  | 150 |
|  | Criterion | entropy |
|  | Max_features | Sqrt |
| Bagging | n_estimators | 100 |
|  | random_state | 42 |
|  | bootstrap | True |
|  | bootstrap_features | false |

# **Text S5. Feature analysis using SHAP-based interpolation and heat map**

Figure S1 shows the SHapley Additive exPlanations (SHAP) analysis-based interpolation for the high contributory features of the training samples. Similarly, Figures S2 (A-B) show the heatmap-based analysis of the encoding schemes versus trained classifiers using predicted Acc and MCC. From the heatmap analysis, we can see that the Acc and MCC of the DNN is high on the Hybrid Features + BTG.


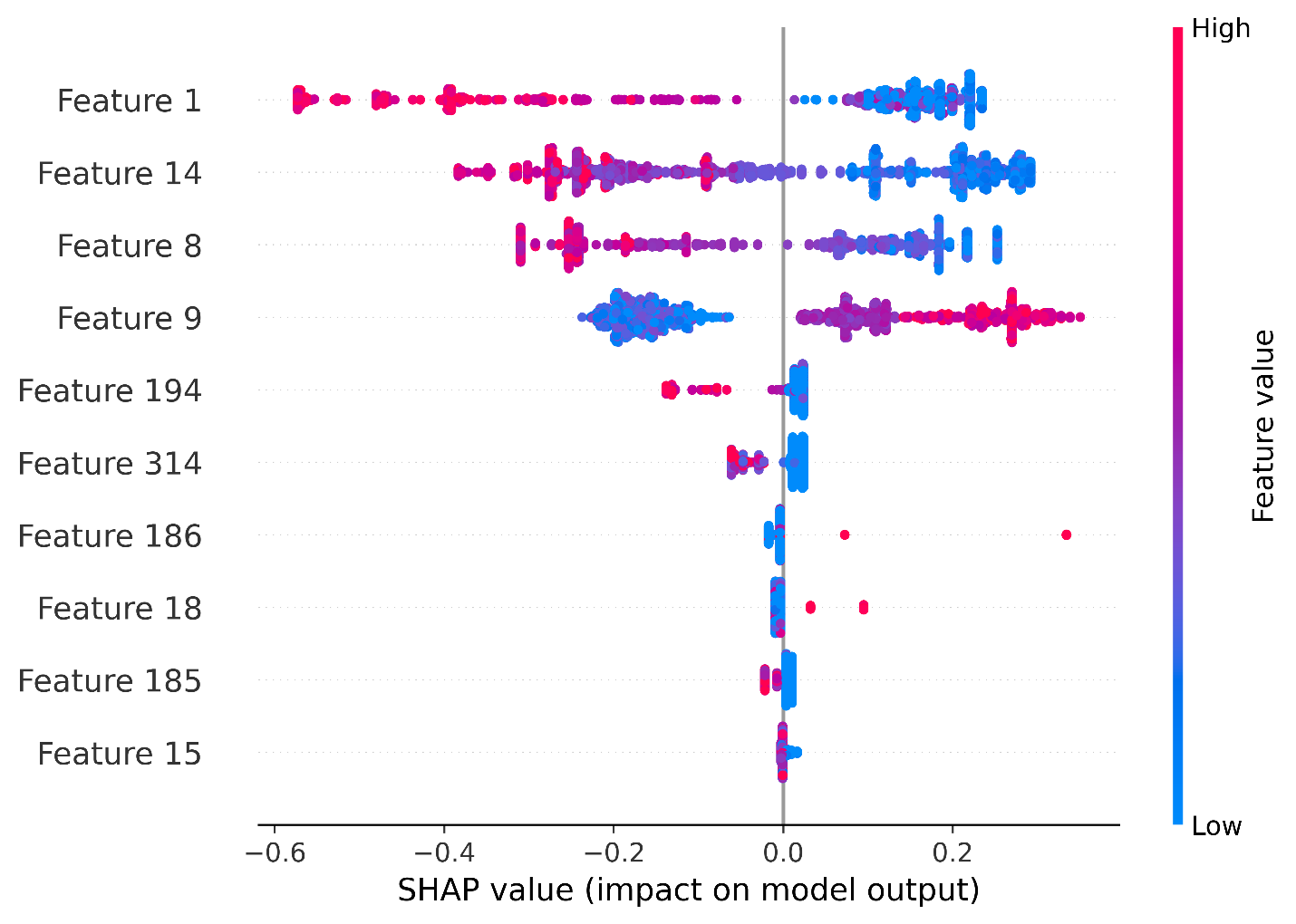


Figure S1. SHAP analysis showing the high contributory features of the training samples.

# **Text S6. Performance comparison with existing methods on the independent datasets**


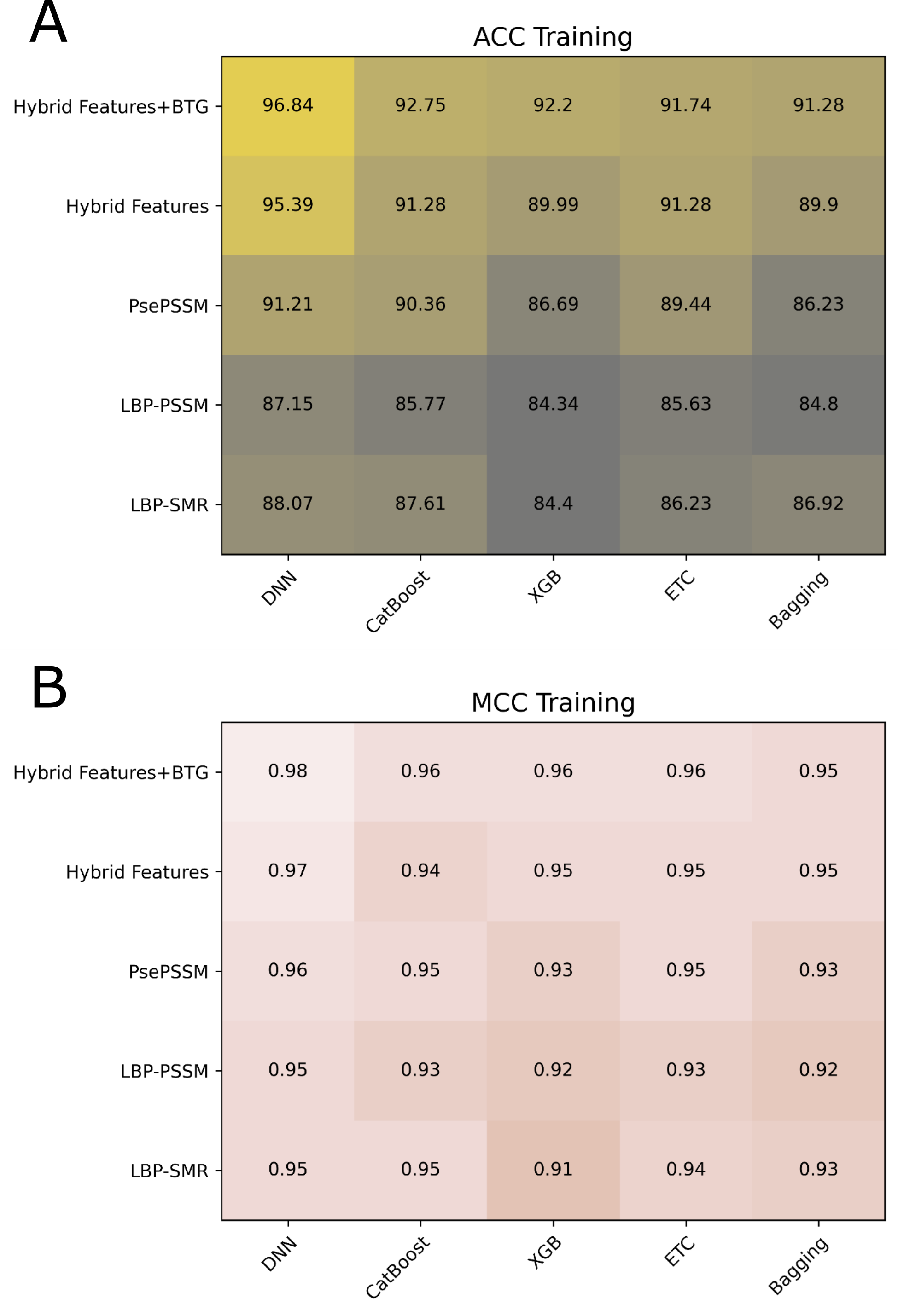


Figure S2. Heat map analysis. Panels (A) and (B) show the heat map of the Acc and MCC, respectively, of the various encoding schemes versus trained classifiers.

The Table S3 shows the results of independent datasets of our method against exisiting state-of-the-art methods in terms of Acc, Sen, Sp and MCC. From the results in Table S3, we can see that, in terms of all performance measures, our proposed DeepAVP-TPPred outperformed all the existing AVP methods in Table S3. In addition, we also performed LIME analysis by taking a random instance from the independent test dataset to validate the proposed model more effectively. The Result of the LIME analysis is provided in Figure S3.

**Table S3.** Performance comparison of DeepAVP-TPPred with existing models using independent test datasets

| **Dataset** | **Predictor** | **Acc (%)** | **Sen (%)** | **Sp (%)** | **MCC** |
| --- | --- | --- | --- | --- | --- |
| **Independent Dataset AVP105** | AVPpred | 85.70 | 88.30 | 82.20 | 0.71 |
|  | Chang et al. | 89.50 | 91.70 | 86.70 | 0.79 |
|  | FIRM-AVP | 92.40 | 93.30 | 91.10 | 0.84 |
|  | Meta-iAVP | 95.20 | 96.70 | 93.20 | 0.90 |
|  | **DeepAVP-TPPred** | **96.09** | **94.83** | **97.33** | **0.92** |
| **Independent Dataset AVP111** | AVPpred | 92.50 | 93.30 | 91.70 | 0.85 |
|  | AntiVPP 1.0. | 93.00 | 87.00 | 97.00 | 0.87 |
|  | Chang et al. | 93.30 | 91.70 | 95.00 | 0.87 |
|  | Meta-iAVP | 94.90 | 91.70 | 98.30 | 0.90 |
|  | **DeepAVP-TPPred** | **95.73** | **95.38** | **97.77** | **0.92** |


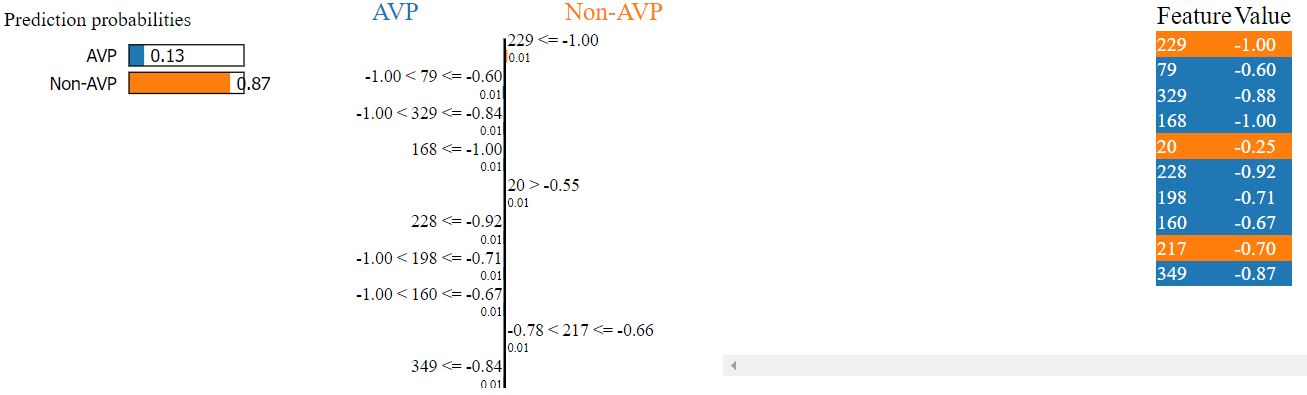


Figure S3. LIME analysis by randomly selected instance from the independent dataset.
